# Changing ideas in forestry: A comparison of concepts in Swedish and American forestry journals during the early twentieth and twenty-first centuries

**DOI:** 10.1007/s13280-015-0744-7

**Published:** 2016-01-07

**Authors:** Erland Mårald, Nancy Langston, Anna Sténs, Jon Moen

**Affiliations:** Department of Historical, Philosophical and Religious Studies, Umeå University, 901 87 Umeå, Sweden; Great Lakes Research Center and Department of Social Sciences, Michigan Technological University, Houghton, MI 49931 USA; Department of Ecology and Environmental Sciences, Umeå University, 901 87 Umeå, Sweden

**Keywords:** Ecology, Forestry concepts, Governance, History, Sweden, The United States

## Abstract

By combining digital humanities text-mining tools and a qualitative approach, we examine changing concepts in forestry journals in Sweden and the United States (US) in the early twentieth and early twenty-first centuries. Our first hypothesis is that foresters at the beginning of the twentieth century were more concerned with production and less concerned with ecology than foresters at the beginning of the twenty-first century. Our second hypothesis is that US foresters in the early twentieth century were less concerned with local site conditions than Swedish foresters. We find that early foresters in both countries had broader—and often ecologically focused—concerns than hypothesized. Ecological concerns in the forestry literature have increased, but in the Nordic countries, production concerns have increased as well. In both regions and both time periods, timber management is closely connected to concerns about governance and state power, but the forms that governance takes have changed.

## Introduction

This study compares the forestry literature of Sweden and the United States (US) during the first decade of the twentieth century and the first decade of the twenty-first century to better understand changing concepts and priorities within the profession. The meanings of forestry concepts are dynamic, changing over time and setting. When we expand our time period and explore the meanings of words in their temporal context, ideas that appear new may actually have longer histories.

A recent analysis (Leipold 2014) of forestry discourse examines 66 published journal articles. Of these, 52 (78.8 %) were quite recent, focusing on the period from 1990 to present. Only four sources examined discourses in the late nineteenth and early twentieth centuries (Leipold 2014). Many studies argue that the broadening of forest management from profit concerns to ecological concerns is a new phenomenon (Farrell et al. 2000; Mather 2001; Veenman et al. 2009). But the short historical timeframe of most studies makes it difficult to know what ideas in forestry are new, and what ideas are actually a reframing of older concerns.

This article examines changing forestry concepts, focusing on Swedish and US journals in the early twentieth and early twenty-first centuries. Although this comparison might be seen as uneven regarding the size, population, and global influence of the countries, when it comes to forestry it is actually a quite even match. In the early twentieth century, Sweden was the largest exporter of sawn forest products in the world, responsible for a quarter of the total world export (Björklund 2000). Today Sweden and the Nordic region are still important within forestry, with the home base for two of the five largest forestry companies in the world (Swedish SCA and Finnish-Swedish Stora-Enso) and the third largest global retailer of sawn forest products (Swedish IKEA) (Dauvergne and Lister 2011).

Other similarities also make the comparison between the US and Swedish forestry literature relevant. Deforestation intensified in both nations at the end of the nineteenth century. These harvests played a key role in the industrialization and modernization of both countries—yet in both nations, deforestation stimulated intense anxieties in the early twentieth century about timber famine, the need for scientific forest management, and the future of the state (Williams 1992; Cox 2010; Antonson and Jansson 2011). Unlike the situation in Germany and France, where professional forestry had been developing for some time, early twentieth century foresters in both Sweden and the United States conceived of themselves as scientific pioneers, facing great challenges in unfamiliar landscapes.

## Historic context and hypotheses

During the eighteenth and nineteenth centuries, modern scientific forestry emerged, although there had been some efforts to systematize forest knowledge into different schemes of management before the Enlightenment (Lowood 1990; Harrison 1992; Radkau 2012). In northern Europe, these developments were linked to the formation of the modern nation-state, as securing control over forests was a way to secure control over the people who used those forests (Warde 2006). Planned felling and replanting over a fixed time were meant to increase control over the balance between yield and regrowth, thus securing a national asset that was vital for energy, industry, transportation, and military state power.

A common characteristic in these efforts was an emphasis on scientific perspectives and professional practices. Foresters tried to gain control over the messy, often chaotic flux of the natural forest by applying theoretical management systems, including even-age monocultures, clear cutting, and regeneration measures (Langston 1995). The objective was to create an improved forest landscape that was predictable in its production of timber or other wood commodities. Such efforts often involved social as well as ecological costs (Guha 1991; Scott 1998). Yet foresters were rarely as rigid or prescriptive as their scientific models suggested. In Europe, diverse ecological circumstances, different cultural understandings, and social conflicts quickly modified the production-focused management agenda (Hölzl 2010). Wherever scientific forest management has been applied, it has met different ecological and cultural settings, sometimes with disastrous results but sometimes involving new learning and the development of new management methods attuned to local conditions (Langston 1995; Grove 1996).

While foresters in Sweden and the US shared many concepts and ideas by the beginning of the nineteenth century, the challenges they faced in implementing those ideas differed significantly. In Sweden, until the late nineteenth century, the authorities were most concerned with maintaining a forest commons, preventing private farmers from overgrazing their forests or from selling their land to timber companies (Ericsson et al. 2000). During the first decade of the twentieth century, professional forest management became established, and foresters began to inventory the forests of Sweden, aiming to transform native forests and marginal farms alike into high-yielding, managed timber stands (Östlund 1995; Eliasson 2011). Demand for pulp, timber, charcoal, and firewood all increased in the first decades of the twentieth century, and by the early 1940s, decreases in timber volume caused great concerns among professional foresters (Ericsson et al. 2000; Lisberg Jensen 2011). In the late 1940s, modern forest management was introduced, including clear-cutting, draining, planting, prescribed burning, scarification, herbicides, and nitrogen fertilization. Since then, the sparsely wooded grazed and burned forest of the nineteenth century has to a large extent been transformed into even-aged forest stands (Ericsson et al. 2000).

In the United States, professional foresters had, as their first task, the administration of public reserves of largely uncut forest, rather than the rehabilitation of marginal farms. American foresters turned to colonial models of forestry, particularly those of British India. Guided by these colonial sources, American foresters understood their task as not just protecting a future timber industry, but more broadly protecting the future of civilization. Shaped by fears of a possible timber famine, US foresters argued that protecting forests was critical for ensuring broader collective goods such as climate amelioration, watershed protection for irrigation, and hydropower—and the basis of civilization itself, which they felt could best thrive in forested landscapes. Although professional foresters had played essentially no role in the day-to-day management of the initial US forest reserves established in 1897, by 1905, when the forester Gifford Pinchot took control of the new US Forest Service, professional foresters managed and administered vast tracts of western forests (Langston 1995; Demeritt 2001). Progressive-era foresters took their tasks quite seriously, deeply concerned about timber famine and its possible effects on society. They turned to the young *Journal of Forestry* to discuss technical management challenges and larger social issues, and also to create an *esprit de corps*—a sense of their identity as forestry professionals.

In both the Nordic countries and the US, professional forestry has found itself challenged during the early twenty-first century by a diverse array of environmental and social groups. Critiques of silviculture and forest management have become common, with scholars arguing that early foresters were concerned primarily with timber production, profit, and state power rather than with ecological functioning and diversity (Alverson et al. 1994; Scott 1998; Puettmann et al. 2009). As historian Donald Worster argues, early American foresters saw themselves as “tree farmers” whose controlling values were efficiency and productivity (Worster 1994, p. 267). Analyses of forest discourses have noted the recent emergence of broader ecological discourses that include awareness of social and ecological contexts (Arts and Buizer 2009; Humphreys 2009; Fischer et al. 2010; Winkel et al. 2011; Leipold 2014; Simonsson et al. 2015). According to these analyses, the forest productionist paradigm that was predominant in the early twentieth century has only recently retreated, replaced by new ecosystem perspectives (Alverson et al. 1994). But when we consider the dynamic meaning of forestry concepts, is it possible that we find a deeper concern with what are now called ecological concepts, such as the effects of forests on soil, water, wildlife, and social conditions? Is it really true that early foresters cared mostly for productivity rather than for broader forest values?

While important similarities exist between Swedish and US forestry, scholars have also noted divergences between Europe and the United States in the development of professional forestry over the course of the twentieth century. Söderqvist (1986) argues that early Swedish foresters were among the pioneer ecologists in Sweden, something that has not been noted for American foresters. Puettmann et al. (2009) state that while European foresters in the early twentieth century were quite attuned to local site conditions, American foresters in the early twentieth century essentially ignored such diversity and tried instead to impose narrow production values on complex forests. Does a broad comparison of the forestry journal collections in both countries support such claims of differences?

We use digital humanities tools to test two hypotheses:

### **Hypothesis 1**

Differences over time: We hypothesize that the forestry literature at the beginning of the twentieth century was more concerned with production and less concerned with ecology than the forestry literature at the beginning of the twenty first century.

### **Hypothesis 2**

Differences over place: We hypothesize that the forestry literature in the United States during the early twentieth century was less concerned with local site conditions than the forestry literature in Sweden at the time.

## Materials and methods

We combine qualitative and quantitative techniques in this analysis. We use digital humanities text-mining tools to compare the relative frequencies of key concepts in US and Swedish forestry journals at two different time periods: in the first decade of the twentieth century, and in the first decade of the twenty-first century. We then use a qualitative approach to examine selected texts and concepts in detail, to better understand their context.

The first investigation period was chosen to provide a long time span which offers a contrast to contemporary understandings and presumptions. Moreover, the early twentieth century was the time when forestry in both Sweden and the US was professionalized and institutionalized, and when the journals we investigate began publication, as described below.

### Data sources

For the Swedish journals, the data used in these analyses include all the articles, editorials, and reviews published during the first ten volume years of *Skogsvårdsföreningens tidskrift* (1903–1912) and in *Scandinavian Journal of Forest Research* (2002–2011). We chose *Skogsvårdsföreningens tidskrift,* which began publication in 1903, for the early period because it was the dominant Swedish forestry journal at the time, including scientific articles, articles by practising foresters, and debates about forests and forest management. The journal included frequently news and reports from other countries around the world. We chose *Scandinavian Journal of Forest Research,* which began publication in 1986, for the later period because it is the closest to a successor to *Skogsvårdsföreningens tidskrift* available. The journal is published in Sweden, and in 2000, over 80 % of the articles had an origin from a Nordic country. This figure declines to about 40 % in 2009 (Hannerz 2010). Therefore, the journal reflects Nordic concerns, but not exclusively. It is a peer-reviewed journal and is consequently more exclusively research-focused than *Skogsvårdsföreningens tidskrift*, yet broader perspectives are reflected in the content, particularly in the editorial content.

For the US, the data used in these analyses include all the articles, editorials, and reviews published in the first ten volume years of *Journal of Forestry* between 1902 and 1912, and during the ten volume years of 2002 and 2011. We chose *Journal of Forestry*, which began publication in 1902 and still continues, because it is the most widely circulated scholarly forestry journal in the United States. Its audience is not just scientists, but forestry managers and professionals. Like the Swedish journals, the *Journal of Forestry* is not exclusively concerned with forests in its country of publication.

### Data manipulation

We downloaded into Zotero (an open-source reference management software^1^) all articles, editorials, and reviews published in the relevant journals. Anonymous pieces were excluded. Where necessary, we used Adobe Professional (an application software to manage files in Portable Document Format, PDF) to perform optical character recognition (OCR). We then extracted all the text from each article. For each country, the year’s articles, editorials, and reviews were combined into a single text file, one for each volume year. We thus created four collections with the following word counts in each collection:

1. Early US: US 1902–1911 (780 441 words)
2. Recent US: US 2002–2011 (2 823 297 words)
3. Early Sweden: Sweden 1903–1912 (2 605 025 words)
4. Recent Sweden: Sweden 2002–2011 (3 432 334 words).

Total text words analyzed: 9 641 097.

### Limitations of the data

OCR resulted in some fragmented words, particularly in collections 1 and 3. Because of the size of the collections, we were not able to manually correct all these fragmented words. The size of the collections, however, means that such fragments should not skew the results appreciably. Comparison across all three journals is not precise, because collection 3 was written in Swedish, and so searches in that collection were done in Swedish. All three journals are broad in scope including both scientific articles, professional recommendations, and debates about forest problems and conflicts. However, as mentioned above, *Scandinavian Journal of Forest Research* focuses more on peer-reviewed scientific literature than the other two journals.

### Analyses

#### Text-mining

We used the open-source program Voyant Tools^2^ to extract relative word frequencies and closely associated terms. Common English and Swedish words (so-called stop words) were excluded from the analysis. Because it was necessary to use different journals for the Swedish and American forestry literatures, we cannot compare *absolute* occurrences of terms across the four collections. However, we can explore differences in *relative* frequencies, which we define as the number of times a given term is used per 10 000 words in that year’s entire collections of texts.

#### Qualitative analyses

For each core theme that was revealed by the text-mining analysis, we also examined quotations from the source material to explore the social and narrative context of these ideas. We selected representative quotations by using data from the text-mining to suggest key words. We then entered those key words into the search function on Voyant-tools that searched all texts in the entire corpus, finding quotations that captured the most important search terms, as indicated by the text-mining data. Text-mining data, in other words, drove the qualitative analyses, rather than the other way around. For the three historians on the team, this was an unusual way of selecting relevant quotations, but we selected this method so that we would not impose our pre-conceived ideas about relative importance of core ideas onto the corpus.

## Results

### Hypothesis 1: Differences over time

We predicted that the forestry literature between 1902 and 1912 would reveal relatively more concern with production factors and less concern with ecological factors, compared to the forestry literature between 2002 and 2012.

#### Forest management for timber

**Fig. 1:**
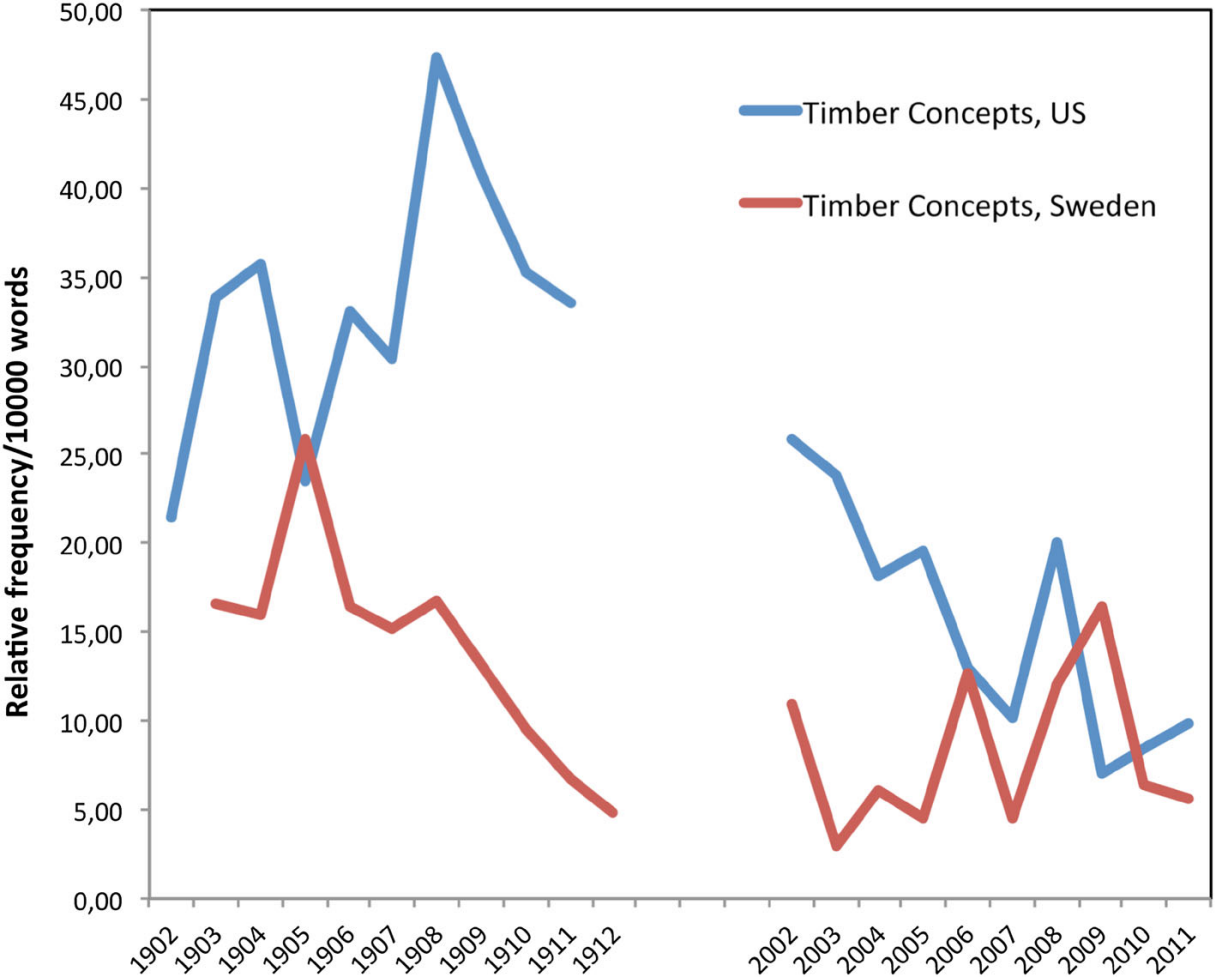
The timber concept

Figure 1 shows differences in the relative frequency of the concept of timber (combining the words timber and lumber) in the US and Swedish forestry collections. For the early Swedish collection, we combined the terms *virke, virket, timmer, virkets, trä*.

These relative frequencies suggest that in both Sweden and the US, early forestry literature was more concerned with concepts related to timber, compared to later Swedish and US forestry literature. Additionally, in both time periods, the US literature seems more concerned than the Swedish literature with concepts of timber and lumber.

**Fig. 2:**
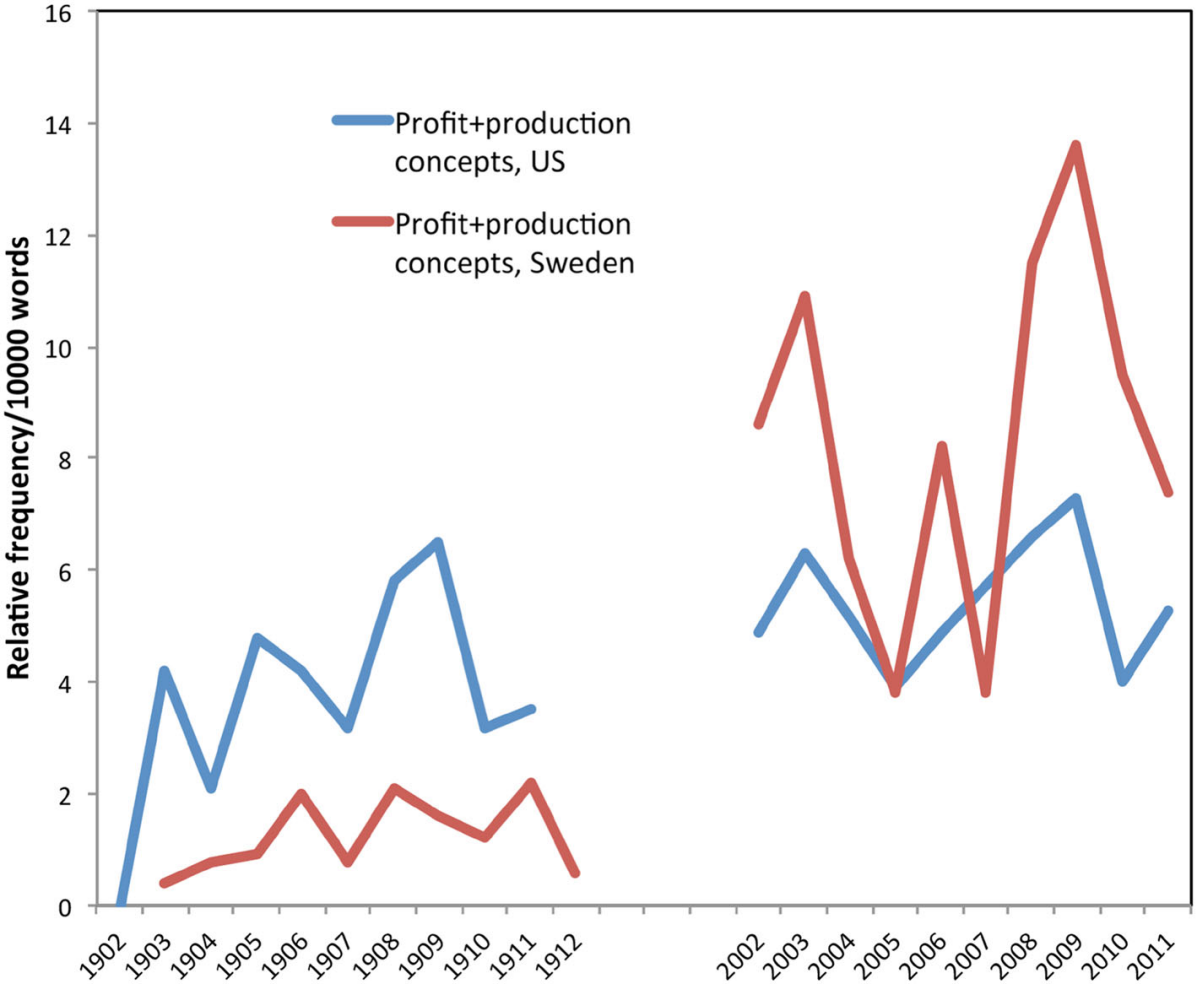
The production concept

In 1912 in the US collection, “timber” was found at a relative frequency of 29.60/10 000 words, while in 2011, the relative frequency was 4.89/10 000 words. Compared to other words in the collections, in the US, “timber” is the fourth most common word in *Journal of Forestry* during the period 1902–1912, with a count *n* = 1760, behind “forest” (*n* = 3962), trees (*n* = 2148), and feet (*n* = 1836). During the later period (2001–2011), “timber” has dropped to the 14th most common word in the journal. Figures 1 and 2 suggest that in the early period, timber was a core concept in forest management discourse in both countries. Timber was clearly viewed from a silvicultural perspective with a distinct goal to increase the utilization of the forest for timber production and economic development. In Sweden, foresters discussed “businesslike management,” and every issue of *Skogsvårdsföreningens tidskrift* contained a section about the market for different forest commodities, including saw timber, pulp, and paper. Similarly in the US, there was a focus on “merchantable timber” and “production of value.”

However, when we look more deeply at the concept of production, we find matters less clear cut. Figure 2 shows the relative frequency of the production concept, which combines the terms profit and production in English, and *vinst* and *produktion* in Swedish. In the US, these terms were not more frequent in the early collection, as we had predicted. This suggests that early American foresters may not have been obsessed with a narrow view of timber production; rather, their understandings of the concept of forest production may have been broader, as discussed below. In the early Swedish literature, the production concept is relatively less frequent than in the early US literature. But in the recent Swedish literature, the relative frequency of the production concept has increased dramatically. It appears that the forestry profession became more focused on fine-tuning specific silvicultural measures to improve forest production. They are also often goal-oriented, where timber is seen as a part of a larger industrial and economical system, sometime called “the forestry production chain.” For example, after the most severe storm in modern Swedish history in 2005, two of the most important Swedish research councils for forest research allocated research funding “to the whole forestry production chain, from silviculture to industrial processing and marketing” (News & Views 2 2005, p. 100).

**Fig. 3:**
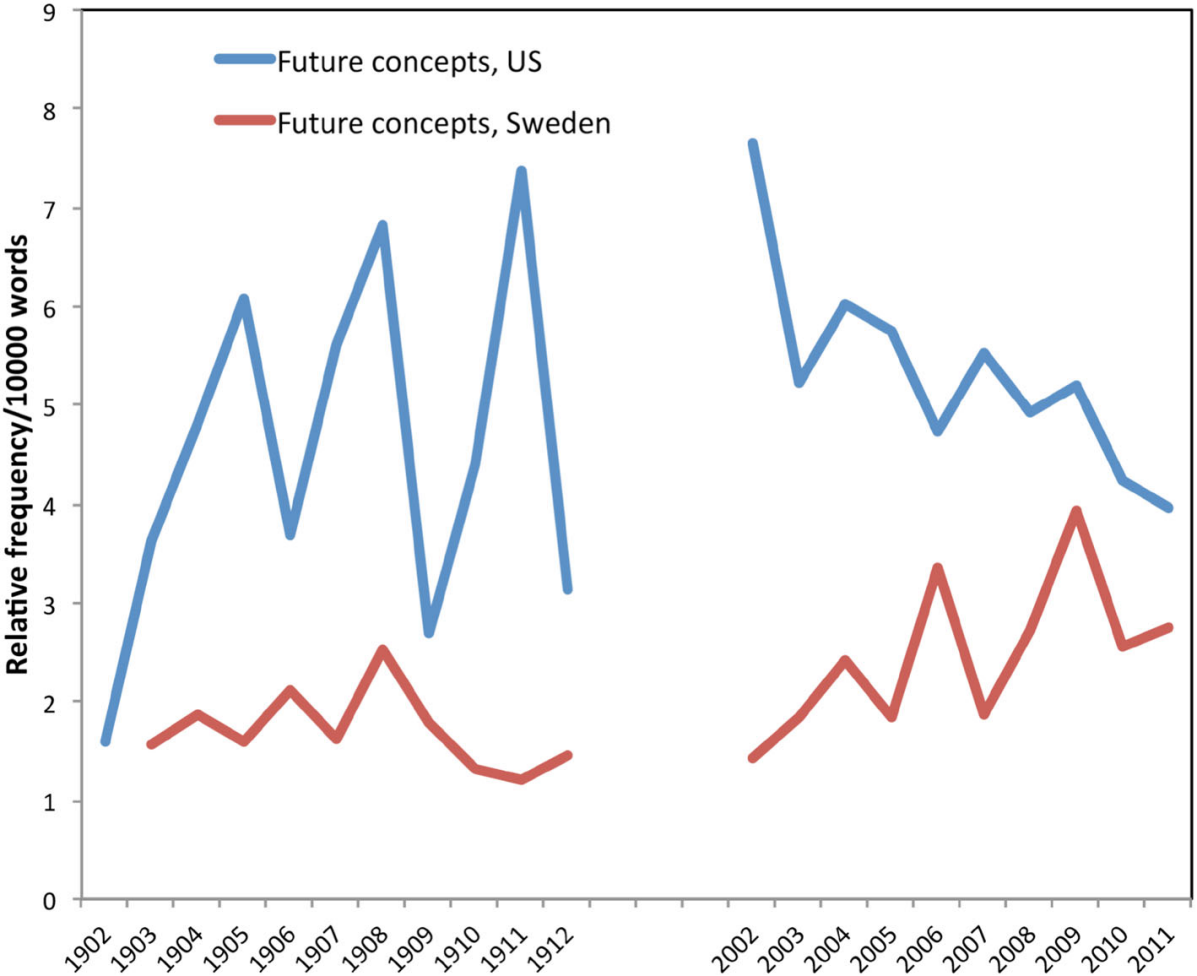
Concerns about the future: relative frequency/10 000 words

Concerns about future forest conditions were critical in motivating early foresters, as scholars have observed (Williams 1992; Cox 2010). But were they equally important in motivating more recent foresters? Figure 3 shows that the concept of the future was stronger in the early American literature than in the early Swedish literature. (US term: “future”; Swedish terms: *framtid*, *framtiden*, and *framtider*). In the later collections, the concept became more frequent in the Swedish literature, and somewhat less frequent in the American literature (so the two nations converged). In all four collections, numerous articles discussed potential threats to future timber production, with a focus on human mis-use.

In the early US forestry literature, concerns about the future focused on the idea of “timber famine” a potential shortage of timber than might devastate the nation. Elliott (1905), for example, writes about the need “to avert the impending calamity of a timber famine” by reforesting the denuded pinelands of Minnesota in the American Midwest (Elliott 1905, p. 100). Such concerns became linked to a call for a specific type of forest governance: public possession of forest lands (rather than control over the actions of private owners on their own lands). As one American forester wrote in 1903, “The timber famine, which the last census shows to be in the near future, …make it imperative that the region be in government possession. It is, however, not only important that the government secures this region, but that when secured be put under proper management,” (News and Notes 1903, p. 76).

Articles in the early Swedish collection expressed fears about a coming “scarcity of forests” caused by two major things: industrial “reckless forest cutting” and “improper” agricultural use by small-scale farmers. The Swedish early literature discussed actions that foresters and the state could take to secure future forests:

The Swedish forest is not yet so close to extinction that one can calculate less than a generation for this point of time. However that may be, let us dedicate ourselves to eliminate those obstacles that exist in our legislation against intensified forest management [*hushållning*], such as enclosure regulations, and to facilitate the stipulations for this kind of management by means of sensible arrangements, in which case our nation, just as before, shortly will find a never ending source of prosperity in its forests. (Zellén 1906, p. 193) [Our translation]

In the early Swedish collection, important ideas included better *“hushållning,”* or rational forest management, implying regulations that would govern private farmers and landowners and thus ensure a future supply of timber and prosperity.

By the early twenty-first century, the primacy of timber in the forest management discourse in the US literature began to decline (Fig. 1), yet concerns about the future persisted. During the so-called “timber wars” of the 1990s and early 2000s, critics challenged the US Forest Service’s focus on timber production and their management of federal lands. As a consequence, the production of timber on national forest land declined sharply between 1987 (26 709 834 m^3^) and 2000 (4 116 563 m^3^)—only 15 % of the peak harvest (USFS 1987, 2000). In 2007, two foresters from the USDA Forest Service wrote in the *Journal of Forestry* that “the days of large-scale timber production on national forest land are gone. There is nothing left to fight about,” (Bosworth and Brown 2007, p. 272). So while concerns about the future hardly vanished in the US literature, these concerns became focused less on timber production, and more on ecological restoration to meet the challenges of changing social conditions and changing climates. As Bosworth and Brown (2007, p. 210) note, “scientists and forest managers have recognized the importance of focusing on healthy ecosystems, and when the main things that Americans want from the national forests and grasslands are clean air and water, habitat for wildlife, and opportunities for outdoor recreation. For a century or more, Americans have drawn down their natural capital on public and private lands alike. It is time to reverse that trend by investing in the forests and grasslands that future generations will depend on for the ecosystem services they need.”

In contrast, the twenty-first century forestry literature in the Nordic countries shows that production-oriented perspectives have intensified. For example, Vierikko et al. (2008, p. 432) note that “Forestry here refers to all kinds of silvicultural activities that are aimed at either enhancing timber production, such as harvesting, drainage, forest road building, shelterwood cutting, or clear-cutting.” However, concern has also increased about other forest products such as biofuels, firewood, and berries. Concerns about the so-called “non-timber forest products” (NTFP) and “non-wood forest products” (NWFP) are present in both twenty-first century collections. For example, one 2009 article in *Scandinavian Journal of Forestry Research* states that “forest planning today is increasingly focused on multiple objective use of the forests…there is a need for models which facilitate the prediction of the impacts of alternative forest management options on non-wood forest products and values so that different aspects of forest use both timber and non-timber can be taken into account in the forest planning process.” (Turtiainen et al. 2009, p. 205). Yet, while perspectives on alternative uses of the forest may have broadened from timber alone, the very terms “non-timber forest products” and “non-wood forest products” suggest that timber remains the norm. One rarely reads about “non-berry forest products” as a way to describe timber production, for example.

#### Forest management for ecological concerns

**Fig. 4:**
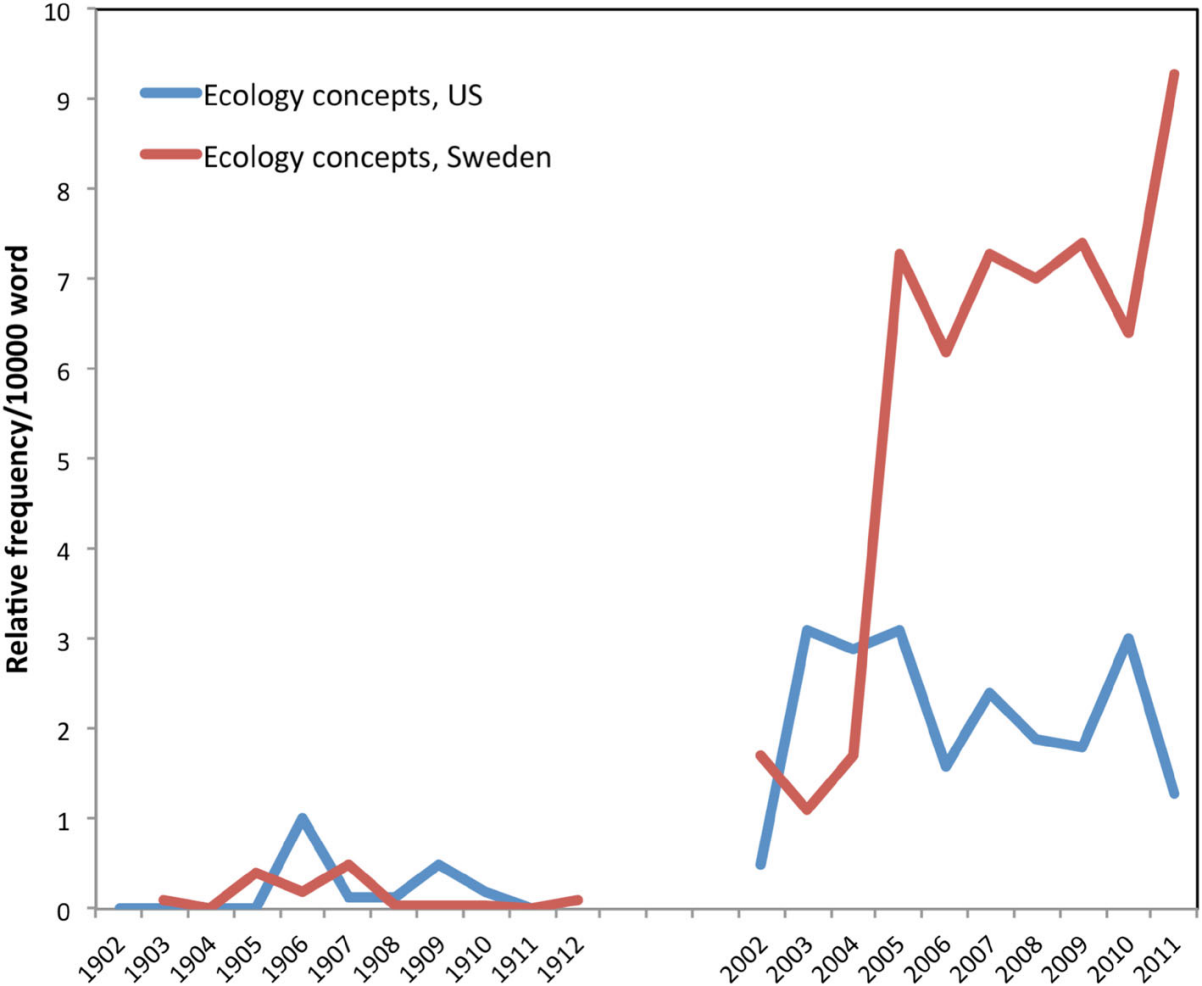
The ecology concept

Did the strong focus on timber production in the first decade of the twentieth century imply a lesser concern with ecological factors? This question is more complicated than it might initially seem. Figure 4 shows that, as predicted, uses of the term “ecology” (in Swedish *ekologi, ekologiska* and *ekologien*) dramatically increased in recent years, with the Swedish literature showing a greater increase than US literature.

However, it is important to note that the low frequency of the term ecology in the early literature does not mean that foresters were uninterested in broader ecological concepts such as watershed protection and wildlife habitat. While the formal term “ecology” had not become a core concept in either nation’s forestry literature in the first decade of the twentieth century, the ideas encompassed by ecology were indeed quite important. Early foresters were interested in forests for much more than mere production or timber.

In Sweden, Söderqvist (1986) argues that the early Swedish forest research network was largely composed of natural scientists who were “pioneer or proto-ecologists,” including Gunnar Andersson, Henrik Hesselman, and Rutger Sernander. These scientists published in *Skogsvårdsföreningens tidskrift* articles on the natural history of forests, exploring invasions of different tree species and evolution of different forest landscapes. The journal also contains several articles dealing with nature conservation, national parks, and wildlife. There is also an awareness of the international development of ecology as a discipline. For example, Hesselman presented 1909 in an article about Charles Darwin, which he saw as the founder of the discipline of ecology:

By his theory of the natural selection Darwin drew the attention of the scientists to the adaptation of the species to the outside world. Both owing to this and to several of his extremely important works Darwin became the proper founder of biology in a limited sense or ecology, the science which deals with the study of how the external and internal structure of organisms depend on the outer conditions under which they exist. In this science, forestry, as far as it concerns the natural phenomena of the forest, is only one aspect. (Hesselman 1909, p. 83). [Our translation]

Similarly, the early US literature also frequently discusses ecological concerns. In 1902, Sterling (1902, p. 18) writes that “we have all learned, it is to be hoped, to look at a forest not as a mere collection of trees, but as an organic whole, the result of actions and reactions of all the factors found within its limits.” Another American forester mentions that not only do forests “serve as a source of timber supply, which is always an important consideration, but, by their location, protect the drainage-basins adjacent to the fertile valleys where successful agriculture is dependent upon irrigation water, and thus they perform another function of the highest utility,” (Sterling 1902, p. 272). Thus, the contemporary concept of “ecosystem services” has its precursors.

**Fig. 5:**
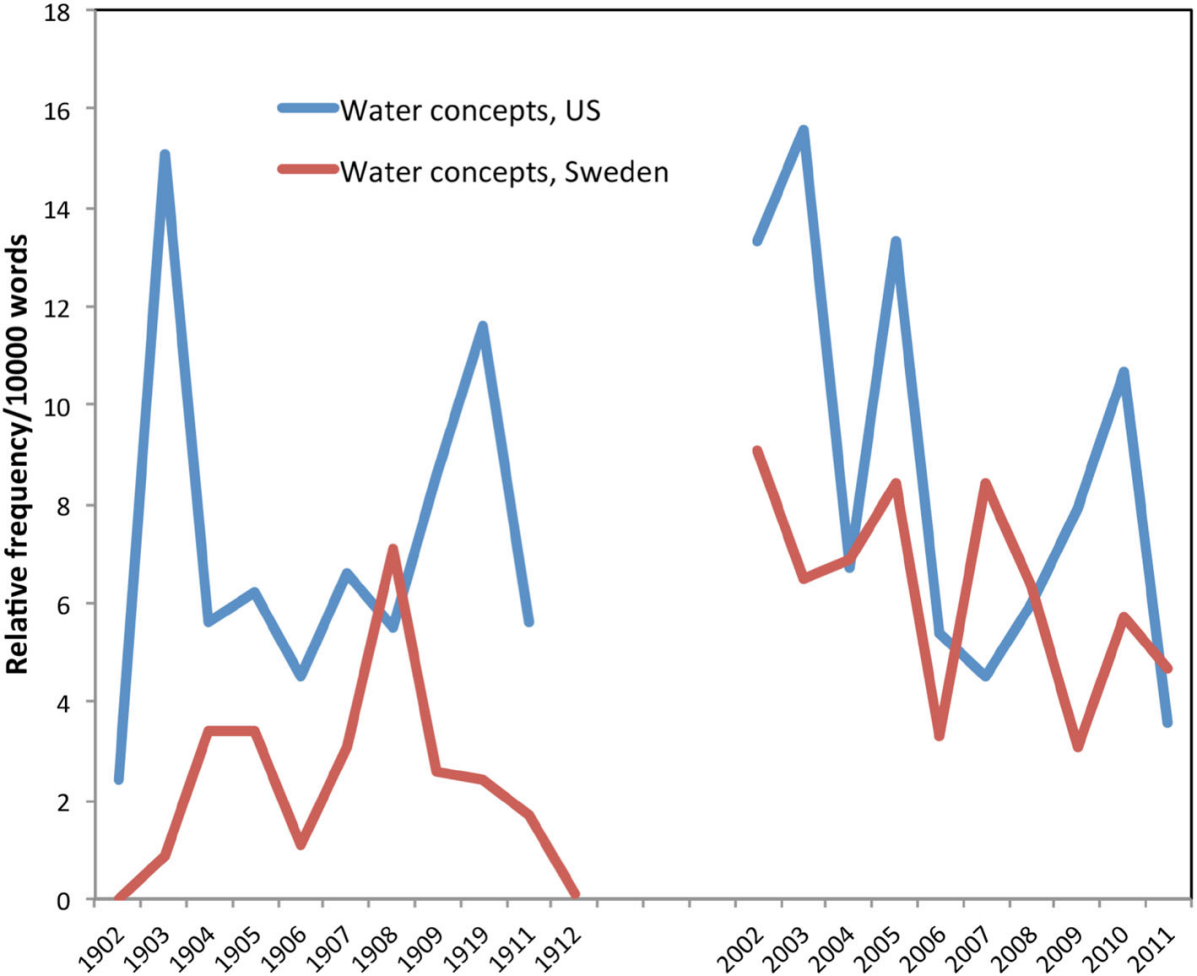
Water and watershed concepts in the literature

As this US quote suggests, water was a core ecological concern of early foresters. Figure 5 shows that the relative frequency of water and watersheds combined (Swedish terms: *vatten, vattendrag, vattendragen, grundvatten, vattendelare, vattenområde, vattensamlingar, grundvattennivå*, and *grundvattennivån*) was actually as strong in the early US collection as in the later collection. This is not surprising, because as Glasser (2005) notes, the founder of American forestry, Gifford Pinchot, “was greatly influenced by [George Perkins] Marsh’s conclusions that civilizations had vanished as a result of abusing watersheds and resources needed for survival.” (Glasser 2005, p. 255). In the relatively arid lands of the US west, concerns about deforestation often focused on the damage to irrigation and hydropower projects. Hodson (1910) discussed a common belief that an “important forest function” was in “rendering the flow of streams more adaptable to economic use,” (160). Hodson states that “the real object of the Forest is to grow the maximum amount of timber…and to protect completely the headwaters of its streams which will be called upon to the maximum for irrigation and for power as the country is developed,” (Hodson 1910, p. 167).

The two nations differed, however, in their concerns about the relations between forests and water. If US foresters were concerned about too little water, Swedes were concerned about too much. Forested wetlands were common in the north of Sweden, and they seemed to impede proper timber production, so the literature extensively discusses the best ways to ditch and drain forests. Even worse was the threat of increasing moisture in Swedish forests. Early Swedish foresters worried about swampification (*försumpning*)—the concern that invading Norway spruce from Finland might make the ground moister and more mossy, thereby reducing areas of productive pine forests (Zellén 1906; Eliasson 2008).

### Hypothesis 2: Differences across place?

#### Forest management in a local ecological context

**Fig. 6:**
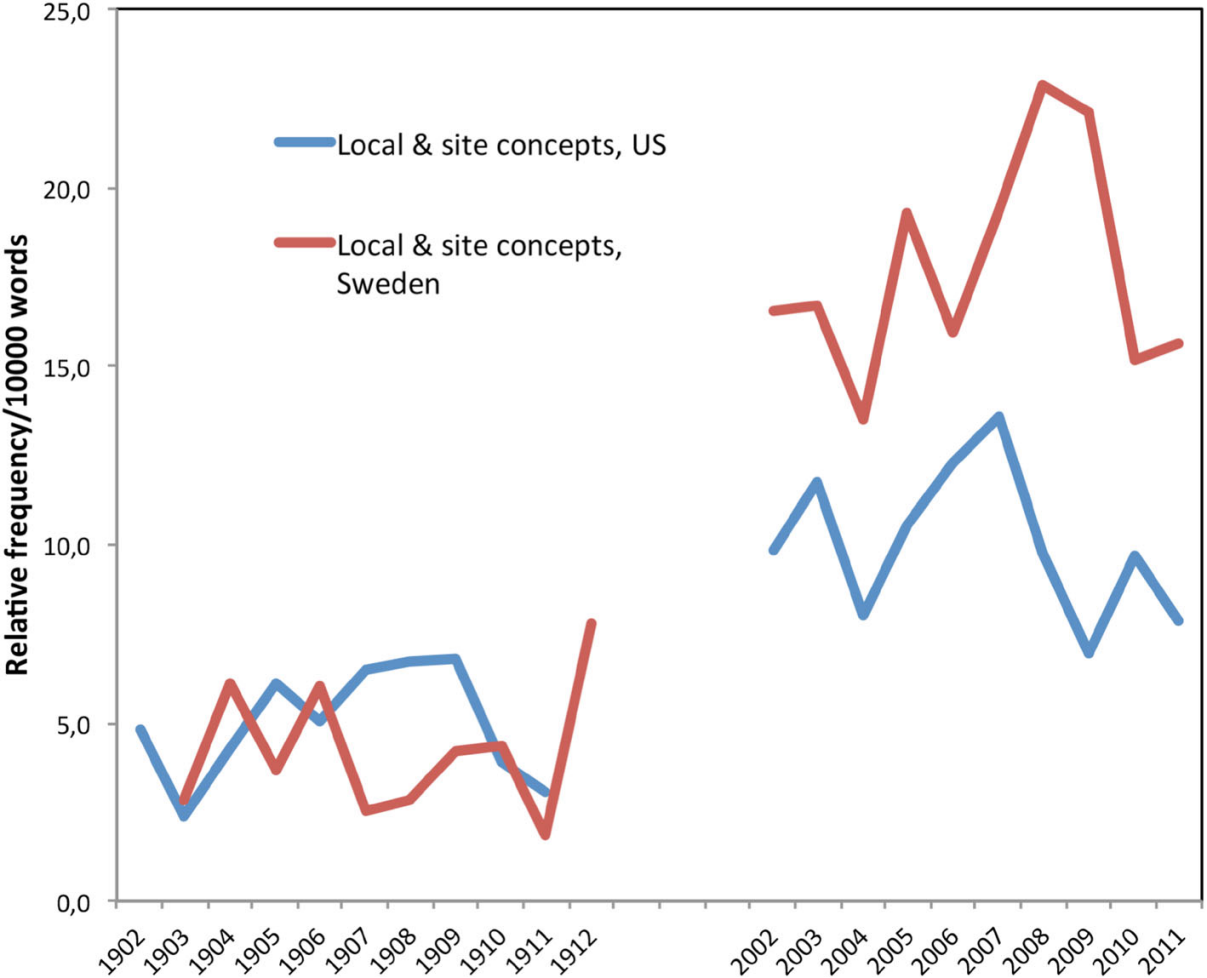
Concerns about local site conditions: the concept of site and local combined

Puettmann et al. (2009) argues that European foresters in the early 20th century were more attuned to local site conditions than were US foresters, who tried to impose uniform objectives on diverse forests. Our collections do not support this argument. Figure 6 examines relative frequencies of local and site combined over time (Swedish terms: *bestånd* and *ståndort*).

Early Swedish forestry literature reveals an awareness about the importance of local conditions, including variations in soil, water, elevation, climate, and wildlife. In part, this awareness may have been influenced by the training of early foresters in Sweden. No doctorates in forestry were conferred in Sweden until 1950. This meant that in the early twentieth century, many of the first forest researchers were recruited from natural science departments, so their training in botany was quite strong. For example Henrik Hesselman, a botanist and a very active author in *Skogsvårdsföreningens tidskrift*, began to work in 1906 at the Swedish forest research institute, and in 1912 he became head of the institute. He specialized in plant geography, plant communities, and the importance of climate and soil conditions. In his view, such ecological understandings allowed one to improve timber production. In 1906 he wrote:

So far in our country, little attention has been paid to the divergent characteristics of forest communities and trees as influenced by differences in our climate. Still, such investigations would have substantial importance as concerns the improvement of our production by means of intensified management. Within each region those trees species and forest communities should be preferred which are best suited to their specific climate. (Hesselman 1906, p. 207). [Our translation]

Early US foresters were as interested in local site conditions as were Swedish foresters. In 1910, the American forester C.D. Mell discussed the “quality of locality,” which meant the “environmental conditions” that determined tree quality (Mell 1910, p. 419). Recknagel and Woolsey (1912) urged young foresters to pay close attention to local site conditions, writing that “A man fresh from his schooling is satiated with theory and requires a year or two of practice. He soon finds that the practice of forestry is limited by what can be done under the local conditions…He must appreciate that nature has the final word and that nature has no exact rules,” (p. 417). But the authors also urged young foresters to study in Europe after a few years of practical experience to gain a better understanding of comparative forestry and European theory—and to keep from becoming “so narrowed that his horizon will be reams of accounts, letters for dictation, and other deadening red tape,” (Recknagel and Woolsey 1912, p. 417).

For both groups, the relative frequency of concerns about local site conditions increases in the twenty-first century, with recent Swedish literature showing more concern than the recent US literature. For example, in Sweden, Pettersson and Högbom (2004) discuss local site conditions in the context of effects of nitrogen fertilization on long-term site productivity. This is mirrored in the domination of a production-oriented management (Fig. 2), although interest in biodiversity and environmental considerations has also increased. In America, being a forester is no longer only a matter of understanding the local ecological conditions; it also requires understanding social and cultural conditions. For example, Knoot and Rickenbach (2011) explore the ways that changing social values in the American midwest may affect the persistence of oak trees across the landscape. As the public becomes uneasy about clearcutting, for instance, oaks may decline as the open-canopy conditions they require for regeneration become locally rarer—for social rather than for ecological reasons alone.

## Discussion

What have we learned by comparing forestry literatures in different centuries and nations? First, we see that early foresters in both countries had much broader—and often ecologically focused—concerns than some scholars have suggested (Alverson et al. 1994; Puettmann et al. 2009). Production and profit have always been important to foresters in both nations, but they have not been the only important concepts. However, the contexts of the concepts have changed over time. For instance, while early foresters were concerned about too much or too little water, contemporary foresters are more concerned about water quality and nutrient leakages (Laudon et al. 2013). In the early decades of forestry, as in recent decades, many competing claims were placed on the forest. Forests today have multiple meanings and multiple uses to broad publics—and the same was true over a century ago.

Important differences do emerge in our analysis, however, across both space and time. In all four collections, timber management is closely connected to concerns about governance and state power, but the forms that governance takes have changed. In both regions, the early period is dominated by a top-down perspective, with centralized experts advising and guiding local practices. The following quote by scientists at the Swedish forest research institute illustrates this perspective: “the individual’s subjective judgement and sympathy for one management method or the other, must be totally subordinated objectively concluded arguments of the experiments,” (Maass 1904, p. 62; our translation). In recent years, however, governing a forest is no longer a matter of applying scientific management principles to various stands; rather, it has become a delicate balancing act between diverse social interests (Knoot and Rickenbach 2011).

While both Sweden and the United States have long had to contend with questions of proper forest governance, the paths they have taken have diverged. In the early collection, foresters in both nations struggled with questions of how best to govern forests across diverse ownerships. In Sweden, the Forestry Law of 1903 focused on the best ways to regulate forestry on private lands. In the United States, however, private forestry was a matter of persuasion rather than regulation. National forestry law focused instead on public lands, where federal foresters could institute scientific management practices without concerns about private property rights (Williams 1992).

These differences reflect broader cultural perspective on private land ownership. Nordic foresters in the early collection primarily discussed how to implement rational forest management on private land, with the guidance of state officials. In the US, foresters had less legal latitude to dictate actions of private landowners on their own properties. The US literature reflects this, with less discussion about forestry on private lands, and more discussion about private trespass on public lands, particularly illegal harvesting and grazing.

Because of the dates chosen for analysis, our study did not throw light on the post-Second World War era, a critical time of change in forestry. Better understanding the reasons for the changes this study has observed would require more attention to the post-World War Two period, which is beyond the scope of this study. As Hirt (1994), Östlund et al. (1997), Cox (2010), and Lisberg Jensen (2011) have all shown, the late 1940s through the early 1960s were years of intense lumber production for both the United States and the Nordic countries. Timber harvests, for example, on the US national forests rose from less than 5 million cubic meters in 1940 to nearly 27 million cubic meters in 1987 (USFS harvest data). In Sweden, the annual harvest on private and public land combined increased from about 70 to 110 million cubic meters between the mid-1950s and 2005 (National Atlas of Sweden 2011). These harvest increases were caused by many factors, including economic growth and construction activity that followed the war. Concerns about harvest practices led to new discourses about ecology, conservation, and endangered species in US forestry (Hirt 1994). Environmental concerns, wilderness protection, and challenges under the Endangered Species Act in the US decreased timber production on public forests in the US since the late 1980s. However, private and industrial forests have continued to be managed primarily for timber production.

So what is actually new in forestry? One way to approach this question is to ask: what ideas have persisted with the end of the timber wars and the rise of ecosystem perspectives? Clearly the idea of management itself persists: the belief that forests need foresters to manage them. The relative word frequency of “management” in all four collections (in Swedish *skogsvård, vård, skogskötsel* and *skötsel*) shows that implementing different measurements in the forest have always been essential, and it is actually something that has become even more important over time. In the recent US literature, “management” is in fact the second commonest word. While management goals have broadened in the twenty-first century, management itself remains the *raison d’etre* among professional foresters. Without doing something active with the forest, the foundation of these professions loses much of its meaning.

## Footnotes

1 http://www.zotero.org.

2 http://voyant-tools.org/.
